# Effect of pH, temperature and freezing-thawing on quantity changes and cellular uptake of exosomes

**DOI:** 10.1007/s13238-018-0529-4

**Published:** 2018-04-03

**Authors:** Yirui Cheng, Qingyu Zeng, Qing Han, Weiliang Xia

**Affiliations:** School of Biomedical Engineering and Med-X Research Institute, Shanghai Jiao Tong University, Shanghai, 200030 China

**Dear Editor,**

Exosomes are cup-shaped small (30–150 nm) extracellular vesicles with the structure of lipid bilayer membrane (Tkach and Thery, 2016) containing proteins, mRNAs and microRNAs that mediate intercellular communication (Valadi et al., 2007). Unlike other extracellular vesicles, exosomes are released into the extracellular space when the multivesicular bodies (MVBs) fuse with the plasma membrane (Colombo et al., 2014). Almost all cell types can secret exosomes and exosomes exist in diverse biological fluids, such as blood, urine, saliva, hydrothorax and breast milk (Thery et al., 2006). Up to now, a number of studies have demonstrated the functions of exosomes in disease development and the potential clinical applications in diagnosis and therapy (Shao et al., 2016). To conduct reproducible studies on exosomal content and function, storage conditions need to have minimal impact on exosomes.

There have been a few studies providing partial confirmation of the effect of different storage conditions on exosomes currently. Using exosomes from urine (Zhou et al., 2006) and conditioned medium (Lee et al., 2016) respectively to investigate the influence of storage temperature on exosomes as measured by Western blot, both groups have concluded that storage below −70 °C for a long time is the best temperature for the recovery of exosomes. On the other hand, Sokolova et al. (2011) applied nanoparticle tracking analysis (NTA) to measure the size changes of exosomes at different temperatures, revealing that storage at 37 °C led to more reduction in exosome sizes than that at 4 °C. However, in this study no information about changes in the particle concentration was reported. Some other studies revealed the effect of pH, storage temperature and cycles of freezing and thawing only on the yield of exosome isolation, but not on quantity changes during storage (Akers et al., 2016; Ban et al., 2015; Zhao et al., 2017). Therefore, the standard criterion of exosomal preservation condition is still undefined.

Herein, we used HEK 293T cells and ExtraPEG method (Rider et al., 2016) to investigate the influence of multiple storage conditions (temperature, cycles of freezing and thawing, pH) on the quantity changes and cellular uptake of exosomes. ExtraPEG is a new polyethylene glycol (PEG) precipitation method for the purification exosomes without affecting their biological activity. Generally, ultracentrifugation (UC) (Mincheva-Nilsson et al., 2016) is most reliable but time-consuming; and precipitation methods such as ExoQuick (patent number: US20130337440 A1) and ExtraPEG can obtain higher yields of exosomes but with impurity of co-precipitated proteins. First, exosomes from the conditioned medium were extracted by ExtraPEG or UC method. After isolation, transmission electron microscope (TEM), NTA and Western blot were performed to analyze exosomes. Exosomes extracted by UC or ExtraPEG were similar in cup-shaped structure (Fig. S1A and S1B), size distribution (Fig. S1C and S1D). And as representative exosome biomarkers, ALG-2-interacting protein X (ALIX), heat shock protein 70 (HSP70) and tumor susceptibility gene 101 (TSG101) were detected in exosomal protein while β-tubulin, widely used as an internal reference to analyze intracellular protein levels, was not detected in exosome samples (Fig. S1E and S1F). These data indicated exosomes were successfully isolated by ExtraPEG method and suitable for the following experiments.

After isolation, the exosome pellets were divided equally into several portions and each portion was stored at different temperatures (−80 °C, −20 °C, 4 °C, 37 °C and 60 °C), or through 1–5 cycles of freezing to −80 °C and thawing, or at different pH levels (pH 4, pH 7 and pH 10). After 24 h, NTA and Western blot were performed to measure the remaining quantity of exosomes. Regarding temperatures, the exosomes stored at 4 °C had the highest concentration (Fig. 1A). Consistent with the NTA results, the exosomes stored at 4 °C showed higher levels of representative exosome markers ALIX, HSP70 and TSG101 (Fig. 1B). With the increasing cycles of freezing and thawing, the exosomal concentration and protein levels of ALIX, HSP70 and TSG101 all decreased (Fig. 1D and 1E). For different pH levels, the loss of exosomal concentration and three exosome markers ALIX, HSP70 and TSG101 at pH 4 and pH 10 was more than that at pH 7 (Fig. 1E and 1F). Interestingly, exosomes stored at pH 4 decreased more sharply than that at pH 10 (Fig. 1F and 1G), suggesting that acidic environment is more destructive for the exosome stability. The size data (Tables S1–3) showed that there was no significant difference in exosome size between different temperature groups, as well as different cycles of freezing and thawing and different pH groups, which indicated that exosomes are not discomposed into smaller or fused into larger vesicles but largely degraded during storage. Next the levels of exosome-associated proteins for the long-term storage at different temperatures (−80 °C, −20 °C, 4 °C) were also detected. The levels of ALIX, HSP70 and TSG101 decreased over time and the degradation rate at −80 °C was less than that at −20 °C and 4 °C (Fig. 1C).

**Figure 1:**
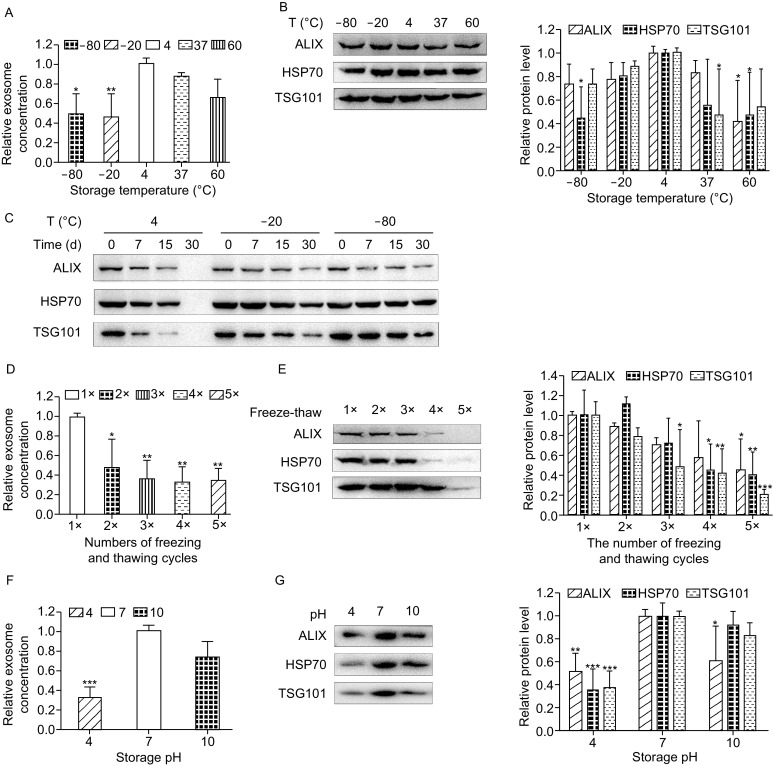
**Effect of different storage conditions on the quantity changes of exosomes**. (A, D and F) The relative concentrations of exosomes stored in different conditions for 24 h were detected by NTA. Bars represent the mean ± SD from at least three independent experiments. (A) Different temperatures. *P < 0.05, **P < 0.01 vs. 4 °C. (D) Different freezing and thawing cycles. *P < 0.05, **P < 0.01 vs. once. (F) Different pH levels. ***P < 0.001 vs. pH 7. (B, E and G) The levels of three exosomal markers, TSG101, HSP70 and ALIX, were detected by Western blot after samples were stored in different conditions for 24 h. The relative quantitation of proteins was shown below the bands. Bars represent the mean ± SD from at least three independent experiments. (B) Different temperatures. *P < 0.05 vs. 4 °C. (E) Different freezing and thawing cycles. *P < 0.05, **P < 0.01, ***P < 0.001 vs. once. (G) Different pH levels. *P < 0.05, **P < 0.01, ***P < 0.001 vs. pH 7. (C) The levels of three exosomal markers, TSG101, HSP70 and ALIX, were detected by Western blot after exosomes stored at −80 °C, −20 °C and 4 °C for 0, 7, 15 and 30 days

**Figure 2:**
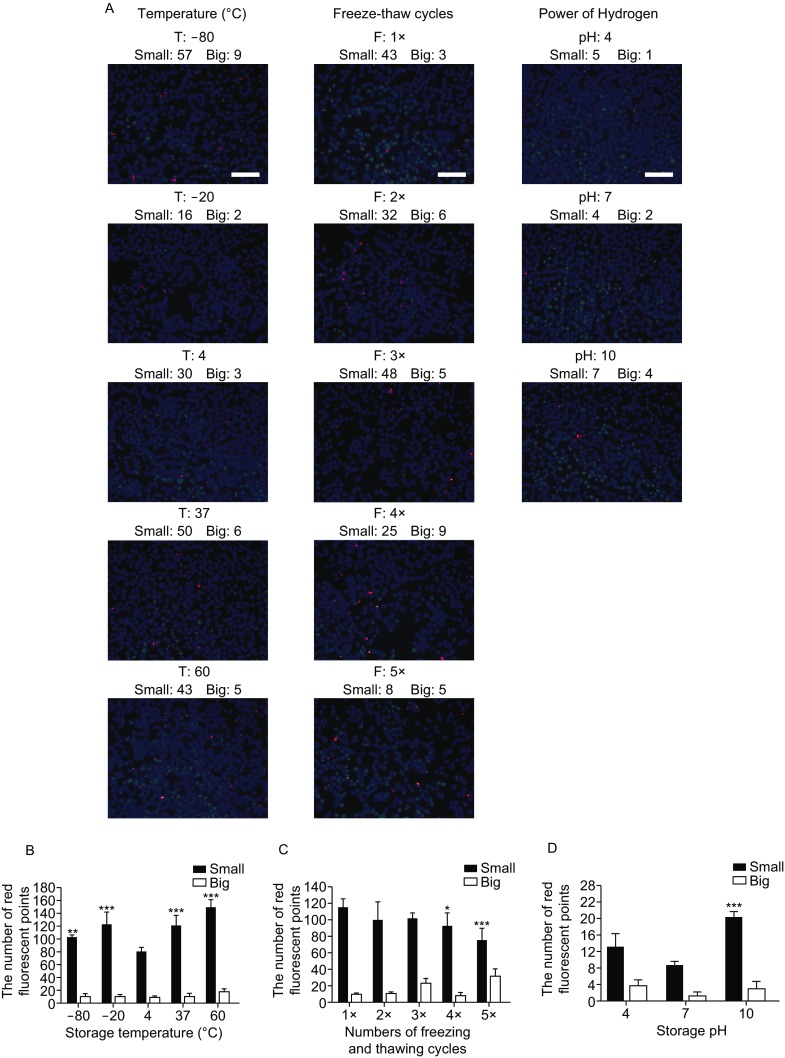
**Effect of different storage conditions on the cellular uptake of exosomes**. (A) PKH26-labeled exosomes stored in different conditions for 24 h and 293T cells were incubated for 3 h. Cell nuclei were visualized by DAPI staining. The numbers of small red spots (smaller than one tenth of the nuclei) and big red spots (bigger than one tenth of the nuclei) were shown above the images. Scale bars: 100 μm. (B–D) The cellular uptake of exosomes was quantified by counting the number of red fluorescent pots. Bars represent the mean ± SD (*n* = 4). (B) Different temperatures. **P < 0.01, ***P < 0.001 vs. 4 °C. (C) Different freezing and thawing cycles. *P < 0.05, ***P < 0.001 vs. once. (D) Different pH levels. ***P < 0.001 vs. pH 7

To investigate the influence of different storage conditions on the cellular uptake of exosomes, the PKH26-labeled exosomes were divided equally into several portions and each portion was stored at different temperatures (−80 °C, −20 °C, 4 °C, 37 °C and 60 °C), or through 1–5 cycles of freezing to −80 °C and thawing, or at different pH levels (pH 4, pH 7 and pH 10). They were then incubated with 293T cells for designated durations. The preliminary experiment showed that 3 h was suitable for the cellular uptake of exosomes; incubating for a longer time increased the aggregations of exosomes and the difficulty in counting (Fig. S2). The fluorescence images (Fig. 2A, all with 3 h incubation) showed that single exosomes (small red spots) and aggregated exosomes (big red spots) were very close to the cell nuclei, which means they are both absorbed by cells (confirmed by confocal microscopy, data not shown). Ignoring aggregated exosomes, there were more single exosomes absorbed by cells after exosomes stored at −80 °C, −20 °C, 37 °C and 60 °C than those at 4 °C (Fig. 2B). And the number of single exosomes reduced with the increasing cycles of freezing and thawing (Fig. 2C), but considering the decrease in exosome concentration, the cellular uptake of exosomes did not have a major change actually. As for pH, storage at pH 4 and pH 10 led to more uptake of exosomes by cells than that at pH 7 (Fig. 2D).

To evaluate the influence of bovine exosomes on the experiments, NTA was performed to measure the concentration of exosomes in 293T cell conditioned medium (50 mL), FBS (50 mL DMEM with 10% FBS), exo-free FBS (50 mL DMEM with 10% exo-free FBS), and exosomes that isolated from exo-free cell medium and stored in different conditions for 24 h. The results showed that exosomes secreted by 293T cells were far more than bovine exosomes in FBS and there were very few bovine exosomes remained in exo-free FBS (Fig. S3A). And the NTA results showed that the effect of different storage conditions on exosomes from exo-free conditioned medium (Fig. S3B–D) was consistent with that from normal conditioned medium (Fig. 1A, 1D and 1F).

It has been commonly recognized that exosomes can be preserved at −80 °C for a long time and higher temperatures over room-temperature (RT) is not suitable for the storage of exosomes (Lee et al., 2016; Zhou et al., 2006). But not much is known about the short-time effect of sub-zero temperatures on exosomes storage. Our results showed the decrease of exosome concentration (Fig. 1A and 1B) and the increase of exosome uptake (Fig. 2B) after storage for 24 h not only happened at 37 °C and 60 °C but also at −20 °C and −80 °C compared with 4 °C. These findings suggested that relatively higher temperature and freezing-thawing cycles could affect exosomal membranes and change their properties so that exosomes could be absorbed by cells more easily. However, further biochemical studies are needed to verify this hypothesis.

Some reports revealed that the acidic pH could reduce degradation of exosome-associated proteins and higher yield of exosome could be isolated after adjustment of pH value to under 7 in conditioned medium or urine and incubation at RT for 30 min (Ban et al., 2015; Zhao et al., 2017). But our results showed that storage at pH 4 decreased the concentration of exosomes (Fig. 1F and 1G) and increases the cellular uptake of exosomes (Fig. 2D). And these findings can be verified by an evidence that doxorubicin loaded in exosomes was released more rapidly and massively at pH 5 than that at pH 7.4 (Qi et al., 2016), and another evidence that increased exosome release and uptake occurred in acidic environment (Parolini et al., 2009). Together, the study of pH influence on exosomes warrants further research.

Our study thus provide relatively comprehensive information on the effects of storage conditions to exosomes, which will be very useful for better preservation of exosomes, and be significant for exosomal function and application studies in future.

## Electronic supplementary material

Below is the link to the electronic supplementary material.

Supplementary material 1 (PDF 308 kb)

Supplementary material 2 (XLSX 9 kb)

Supplementary material 3 (XLSX 9 kb)

Supplementary material 4 (XLSX 9 kb)
