# Supplementary material for: Effect of pH, temperature and freezing-thawing on quantity changes and cellular uptake of exosomes
Source: Protein Cell. 2018 Apr 3;10(4):295–9. doi: 10.1007/s13238-018-0529-4 (PMC6418301; doi:10.1007/s13238-018-0529-4)
Supplement: Supplementary file 1 — Supplementary material 1 (PDF 308 kb) [file 13238_2018_529_MOESM1_ESM.pdf]

Materials and Methods

Cell culture

Human embryonic kidney (HEK) 293T cells were from American Type Culture Collection (ATCC) (Manassas, VA, USA). Cells were maintained in Dulbecco's Modified Eagle's Medium (DMEM; HyClone, SH30243.01) containing 10% fetal bovine serum (FBS; LONSA, S711-001S) or exo-free FBS and 1% Penicillin-streptomycin (PS; HyClone, SV30010) at 37℃ in a humidified incubator (Thermo, Forma Series II) containing 5% CO_2. Cells were cultured for 1-2 days prior to collecting the media containing secreted exosomes.

To deplete bovine exosomes from FBS, FBS was centrifuged at 120000 g for 16 h at 4℃ and supernatant was exo-free FBS.

Isolation of exosomes

Exosomes were isolated using an ExtraPEG method (Rider et al., 2016). Briefly, normal or exo-free conditioned media harvested from cell culture was first centrifuged for 5 min at 500 g immediately to remove cells and large debris. The supernatant was then centrifuged at 2000 g for 10 min to remove large vesicles. Once centrifuged, the media was mixed with 16% polyethylene glycol (PEG; Sigma, 81260) solution by 1:1 ratio to achieve a final PEG concentration (8%) and incubated at 4℃ for over 12 h. After incubation, the mixture was centrifuged at 3210 g (Beckman, Allegra X-15R) for 1 h at 4℃. The supernatant was removed and the resulting pellet

was then suspended in 5 mL PBS (HyClone, SH30256.01). The sample was further purified by adding 5 mL (1:1 to PBS) 10% PEG solution (final concentration is 5%) and centrifuged at 3210 g for 1 h at 4℃ for a second time. The remaining exosome pellet was used for subsequent experiments.

To verify that ExtraPEG method is comparable to UC method, exosomes were also extracted using UC method and 30% sucrose cushion (Mincheva-Nilsson et al., 2016). Briefly, conditioned media was first centrifuged for 30 min at 2500 g, followed the supernatant was centrifuged at 10,000 g,4℃ for 35 min. Then, the supernatant was transferred into 38.5 mL ultracentrifuge tubes (Beckman, 326823) and 4 mL 30% sucrose cushion was added into the bottom of each tube. Finally, the tubes were centrifuged for 70 min at 100,000 g, 4℃ in an SW 32 Ti swinging-bucket rotor (Beckman). To remove sucrose, about 3.5 mL sucrose fraction from the bottom of each tube was collected and diluted with PBS. Then the solution was ultracentrifuged for 70 min at 100,000 g again. The supernatant was discarded and the exosome pellet was resuspended in 1 mL PBS for TEM.

Exosomes storage conditions

The exosome pellet was suspended in a proper volume of PBS and the resulting suspension was then equally divided into five portions. As for different temperature, the five samples were stored for the same time at -80℃, -20℃, 4℃, 37℃ and 60℃. As for different freezing-thawing cycles, the samples were stored at -80℃ and then thawed, followed by 0-4 additional cycles of freezing and thawing. As for different

pH levels, the samples were suspended in a proper and same volume of PBS at pH4, pH7 or pH10, respectively. Then the three samples were stored at 4℃ for the same time (24 h). pH in PBS was adjusted with hydrochloric acid (HCl; Hushi, CHN, 10011018) or sodium hydroxide (NaOH; Hushi, CHN, 10019718) solution and measured by a pH meter (Mettler Toledo, FE20).

Transmission electron microscopy (TEM)

Following storage in different conditions, the samples were prepared for electron microscopy imaging (TEM; FEI, Tecnai G2 spirit Biotwin) based on Thery's method (Thery et al., 2006). Briefly, the exosomes (5 μL) was dripped onto a copper grid (Zhongjingkeyi, CHN, BZ110223b). After one minute of sedimentation, the droplet was sucked out using the air-laid paper. Then the 2% uranyl acetate (Merck, 1.01005.9025) solution (5 μL) was dripped onto the same copper grid for negative-staining and sucked out again one minute later.

Nanoparticle tracking analysis (NTA)

To determine the concentration changes of exosome samples stored in different conditions, the NTA measurement was performed using the ZetaView instrument (Particle Metrix, Germany). The samples were diluted in PBS at proper ratios to achieve the optimal detectable concentration (about 10^7 particles per mL). For each sample, 3-5 mL of the diluted sample was injected into the cell and then the concentration of the sample as well as the mean and median size was determined by

the software (ZetaView 8.03.04.01).

Western blot

The samples were divided into equal parts before their storage in different conditions. Western blotting was performed using a standard method (Shao et al., 2016a) to detect the multiple protein content changes of the samples after their storage. Briefly, the protein concentration was measured using the BCA assay kit (Thermo Scientific, Rockford, IL, USA). The extracts containing 20-30 μg of total protein were separated by 10% SDS-polyacrylamide gelelectrophoresis (SDS-PAGE; EpiZyme, PG112), and were electrotransferred onto a nitrocellulose membranes (GE Healthcare, 10600002) using a semi-dry electrotransfer unit (Trans-Blot SD semi-dry transfer cell, BioRad, Hercules, CA, USA) according to the instrument protocol. The membrane was blocked with 5% nonfat milk powder suspended in tris-buffered saline and tween 20 (TBST) and hybridized with corresponding primary antibodies: TSG101 (Abcam, Cat.# 133586), HSP70 (Cell Signaling Technology, Cat.# 4872S), and ALIX (Cell Signaling Technology, Cat.# 2171S), β-Tubulin (Abcam, Cat.# 6046). After washing with TBST, protein bands specific for the antibody were visualized by enhanced chemiluminescence (ECL; Thermo Scientific, Rockford, IL, USA) and images were captured using chemiluminescent imaging system (Tanon, 5200, China).The intensities of the bands were quantified using ImageJ software.

Exosome labelling

Exosomes were isolated from exo-free conditioned medium using an ExtraPEG method. Purified exosomes were labelled with a PKH26 fluorescent labelling kit (MINI26-1KT; Sigma, SLBS9335) according to the manufacturer's recommendations. Briefly, exosomes were resuspended in 1 mL Dilution C and 1 μL PKH26 was diluted in 1 mL Dilution C. Then, the 1 mL resuspended exosomes was mixed with 1 mL diluted PKH26 and incubated for 1 min. After that, the reaction was stopped with 2 mL added exo-free FBS and exosomes were washed once with PBS at 100000 g for 70 min at 4℃. The labelled exosomes were used for subsequent experiments.

Exosome uptake assay

293T cells were seeded on coverslips in 24-well plates and then labelled exosomes were added to exo-free culture medium. After 3 h, 6 h or 9 h, cells were washed with PBS and fixed in 4% paraformaldehyde for 10 min. Cells were subsequently washed third with PBS, followed by incubation with 0.5% DAPI for 5 min. And then cells were washed third with PBS again and slides were mounted with coverslips. Finally, epifluorescence images were taken using a laser-scanning microscope (Leica TCS SP5 II, Germany)

Statistical analysis

Each experiment was repeated at least three times. Data were analyzed and all statistical graphs were generated by GraphPad Prism 6.0 (GraphPad Software Inc, La Jolla, CA, USA). Differences between two groups were analyzed using one-way or

two-way ANOVA and the probability value below 0.05 was considered significant.

Figure S1. Characterization of exosomes isolated from conditioned medium of 293T cells. (**A-B**)The morphology of exosomes was detected by TEM. Scale bars: 200 nm. (**C-D**) The particle size distribution of exosomes was measured by NTA. **C.** Mean size: 120.5 nm. Median size: 115.3 nm. **D.** Mean size: 146.5 nm. Median size: 133.3 nm. (**E-F**) The levels of exosome-associated proteins, TSG101, HSP70 and ALIX, from cells and isolated exosomes were detected by Western blot. The blots were reprobed for β-Tubulin as a loading control.

Figure S2. The uptake of exosomes by 293T cells after incubation for 3 h, 6 h and 9 h. 293T cells were treated with PKH26-labeled exosomes for 3 h, 6 h and 9 h. Cell nuclei were visualized by DAPI staining. Exosomes with no PKH26 staining were used in control group. Scale bars: 100 μm.

Figure S3. The results were not affected by exosomes from FBS (Refer to Figure 1). (**A**) The concentrations of exosomes from cell medium, FBS and exo-free FBS were detected by NTA. Bars represent the mean ± SD from at least three independent experiments. *$p<0.05$ *vs*. FBS. (**B-D**) The concentrations of exosomes from exo-free cell medium were detected by NTA after samples were stored in different conditions for 24 h. **B.** Different temperatures. **C.** Different freezing and thawing cycles. **D.** Different pH levels.

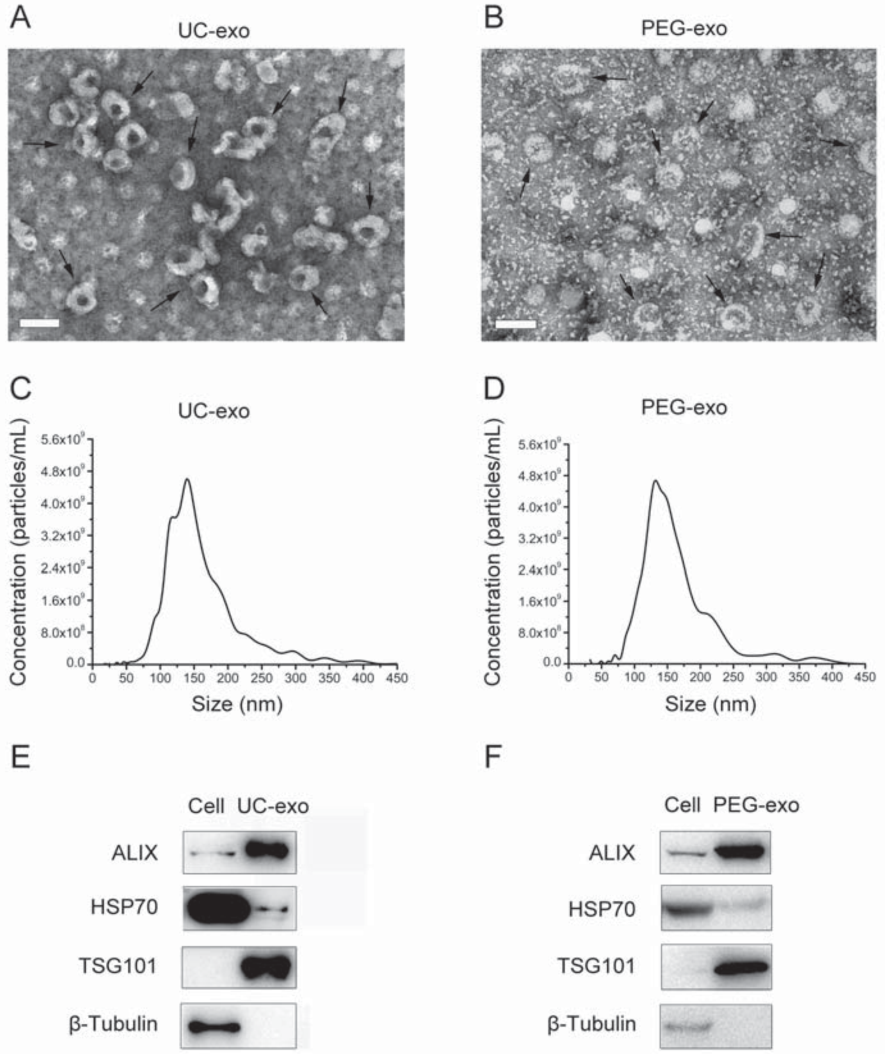
A
UC-exo
B
PEG-exo
C
UC-exo
D
PEG-exo
Concentration (particles/mL)
Size (nm)
E
Cell
UC-exo
ALIX
HSP70
TSG101
β-Tubulin
F
Cell
PEG-exo
ALIX
HSP70
TSG101
β-Tubulin

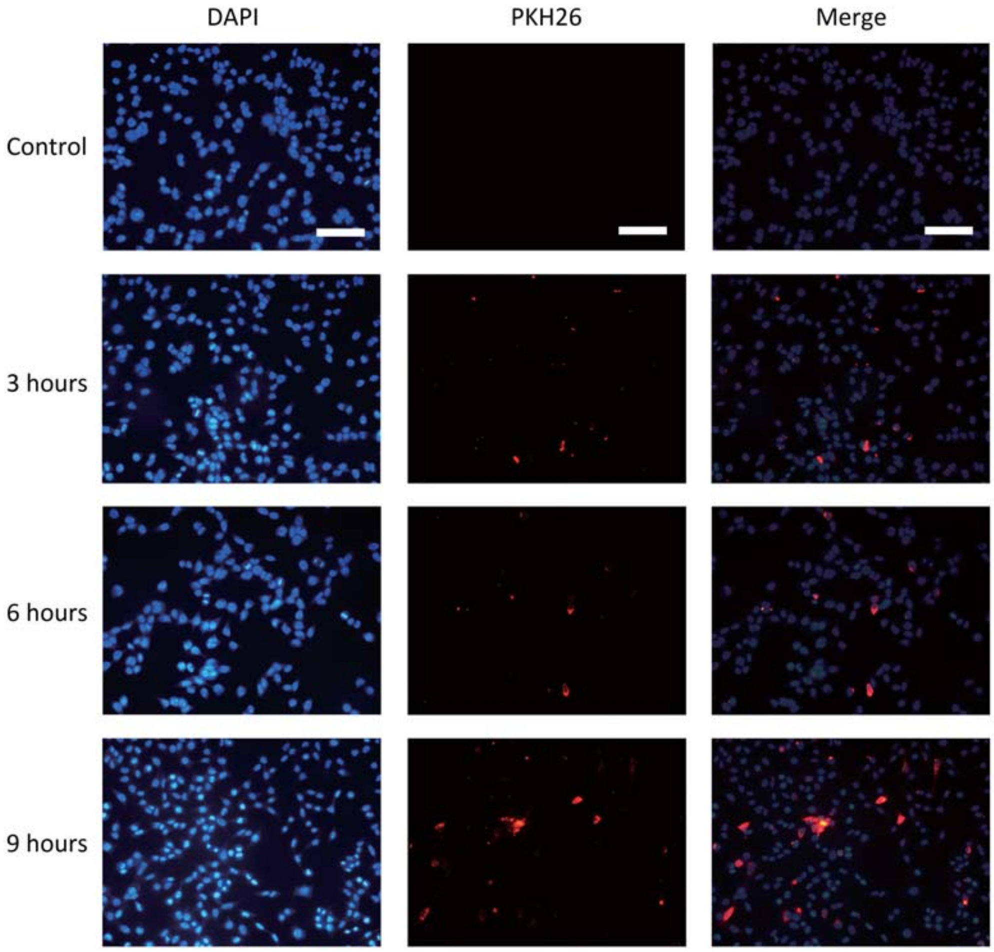
DAPI
PKH26
Merge
Control
3 hours
6 hours
9 hours

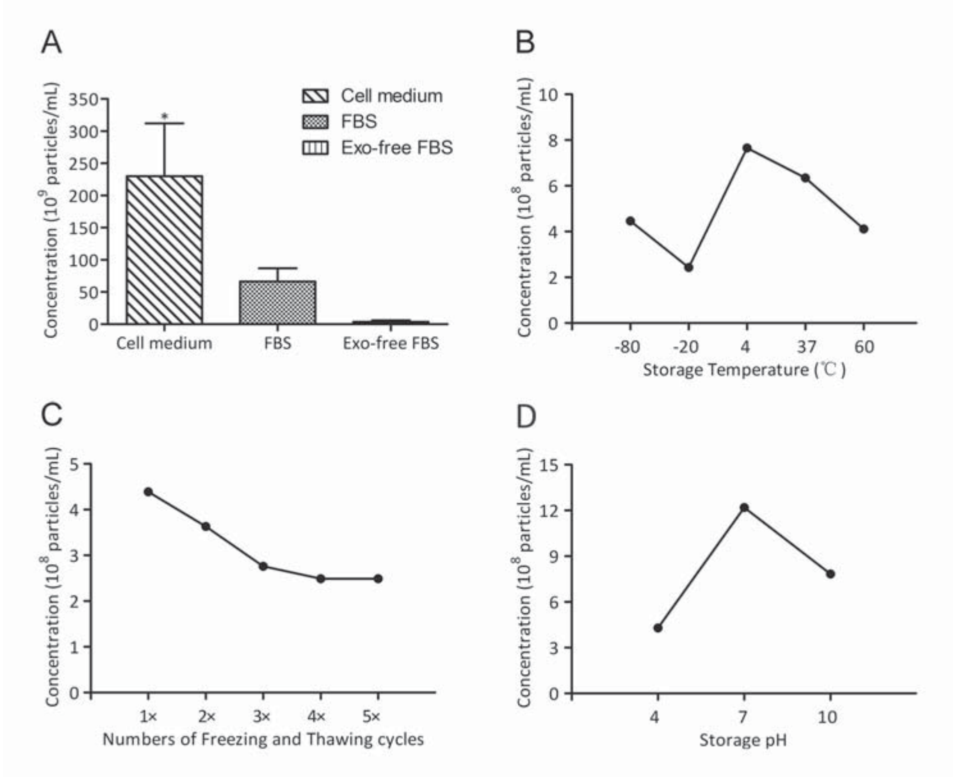
A
Concentration (10^9 particles/mL)
350
300
250
200
150
100
50
0
*
Cell medium
FBS
Exo-free FBS
B
Concentration (10^8 particles/mL)
10
8
6
4
2
0
-80
-20
4
37
60
Storage Temperature (℃)
C
Concentration (10^8 particles/mL)
5
4
3
2
1
0
1×
2×
3×
4×
5×
Numbers of Freezing and Thawing cycles
D
Concentration (10^8 particles/mL)
15
12
9
6
3
0
4
7
10
Storage pH
